# The YAP1/GPX4 axis alleviates osteoporosis by affecting ferroptosis in osteoblasts

**DOI:** 10.1186/s10020-025-01374-4

**Published:** 2025-10-21

**Authors:** Mingsi Deng, Yong Zhou, Gengyan Liu, Ruimin Tang, Liangrong Hu, Jia Luo, Zhipeng Tang, Liangjian Chen, Zhengguang Wang

**Affiliations:** 1 Department of Stomatology, The Third Xiangya Hospital of Central South University, 138 Tongzipo Road, Yuelu District, Changsha City, Hunan Province P.R. China; 2 Department of Orthodontics, Changsha Stomatology Hospital, Changsha City, Hunan Province P.R. China; 3 Department of Spinal Surgery, The Third Xiangya Hospital of Central South University, 138 Tongzipo Road, Yuelu District, Changsha City, Hunan Province 410013 P.R. China; 4 Department of Orthopedics, The Third Xiangya Hospital, Central South University, Changsha City, 410013 Hunan Province P.R. China; 5 Department I of Orthopedics, Wugang Light Textile Orthopedic Hospital, Wugang City, Hunan Province 422400 P.R. China; 6 Changsha Blood Center, Changsha City, Hunan Province 410000 P.R. China; 7 Jinshi Hospital of Traditional Chinese Medicine, Changde City, Hunan Province 415400 P.R. China

**Keywords:** GPX4, YAP1, Ferroptosis, Osteoblasts, Osteoporosis

## Abstract

**Background:**

Osteoporosis (OP) is a disease in which weak bones increase the risk of fracture. It has been reported that the occurrence of ferroptosis accelerated the progression of OP. However, the underlying mechanism of ferroptosis in OP remains unclear.

**Methods:**

Clinical samples from OP patients were collected and ovariectomized (OVX)-induced mouse models with GPX4 knockout was established. The expression of genes and proteins was determined by RT-qPCR, western blot, IHC and IF. Bone mineral density (BMD) of the lumbar vertebrae was evaluated using DXA. Pearson correlation analysis was used to analyze the relationship between GPX4 expression and BMD. The femoral morphology was detected by HE staining. Images and relevant parameters of the femur were acquired using micro-CT. Ultrastructural changes in mitochondria were observed using TEM. MDA and GSH levels in mice and cells were examined using commercial kits. Lipid peroxidation was detected using Bodipy-C11 fluorescent probe. ALP activity was measured using ALP staining and calcified nodules were examined using ARS staining. The interaction between YAP1 and GPX4 promoter was validated using ChIP and dual-luciferase reporter gene assay.

**Results:**

GPX4 expression was downregulated in clinical samples of OP and positively correlated with BMD. GPX4 knockout exacerbated bone loss and promoted ferroptosis in OVX-induced mice. Besides, GPX4 overexpression inhibited ferroptosis and enhanced osteogenic potential of osteoblasts. Moreover, YAP1 positively regulated GPX4 expression in osteoblasts through activating transcriptional activity of GPX4 promoter and YAP1 overexpression suppressed ferroptosis and enhanced osteogenic potential of osteoblasts via enhancing GPX4 expression.

**Conclusion:**

GPX4 was positively regulated by YAP1, which in turn inhibited ferroptosis and enhanced osteogenic potential of osteoblasts, thereby alleviating OA progression.

**Supplementary Information:**

The online version contains supplementary material available at 10.1186/s10020-025-01374-4.

## Introduction

Osteoporosis (OP) is a progressive systemic bone disorder characterized by reduced bone mass, impaired bone microstructure, decreased bone quality and increased fracture risk. It has been recognized as a public health issue affecting the health of middle-aged and elderly populations (Compston et al. 2019; Eastell et al. 2016). Currently, clinical management of OP remains dominated by pharmacological interventions, including bone formation-promoting agents, bone resorption-inhibiting agents, calcium and vitamin D supplements and traditional Chinese medicines (Khosla and Hofbauer 2017; Lei et al. 2021). However, various drugs are associated with significant adverse effects and limitations(Rossini et al. 2016). Therefore, exploring the pathological mechanism of OP and identifying novel therapeutic targets is of considerable research value.

Ferroptosis is an iron-dependent form of programmed cell death distinct from apoptosis, autophagy, and necrosis. It is characterized by the inhibition of glutathione peroxidase 4 (GPX4) and System Xc^−^, which leads to disrupted cysteine metabolism and enhanced lipid peroxidation (Yang and Stockwell 2016). Numerous studies have indicated that ferroptosis is widely involved in various diseases, and has emerged as a research hotspot across multiple fields in recent years(Stockwell et al. 2020). Currently, massive evidences have demonstrated a close connection between ferroptosis and OP progression, supporting that that suppressing ferroptosis could alleviate OP progression (Gao et al. 2022a, b; Jing et al. 2023). In this study, we aim to identify crucial molecules underlying the regulation of ferroptosis in OP.

As is well-known, GPX4 plays a suppressive role in ferroptosis, which eliminates lipid peroxides by reducing them to lipid alcohols, thereby preventing the accumulation of lipid reactive oxygen species in the body (Seibt et al. 2019). Indeed, studies have reported that GPX4 was closely implicated in OP progression. As previously documented, activation of ferroptosis process was found in rats with diabetic OP, accompanied by decreased GPX4 expression (Lin et al. 2022). Exosomes derived from vascular endothelial cells suppressed ferroptosis and elevated GPX4 expression, thereby impeding OP progression (Yang et al. 2021a, b). Additionally, GPX4 mitigated steroid-induced OP in mice by suppressing ferroptosis (Lu et al. 2019). The above evidence indicated that GPX4 plays a crucial role in OP. However, the specific functions and mechanisms of GPX4 in OP remain unclear and warrant further investigations.

The Hippo signaling pathway is an evolutionarily conserved signaling cascade that regulates multiple biological processes, such as cell growth, organ size control, and regeneration (Ma et al. 2019). Studies have reported that it also played an important role in OP (Li et al. 2023a, b). As a Key transcriptional coactivator and the terminal effector in the Hippo signaling pathway, Yes-associated protein 1 (YAP1) is a key molecule involved in the regulation of various diseases, including gastric adenocarcinoma, cardiomyocyte hypertrophy and OP (Ajani et al. 2021; Yue et al. 2022; Zhong et al. 2021). A previous study revealed that YAP1, regulated by circ-ITCH/miR-214 axis, promoted osteogenic differentiation to ameliorate OP (Zhong et al. 2021), indicating that higher YAP1 expression was conductive to preventing OP progression. Notably, evidence has suggested that YAP1 could exert a suppressive effect on ferroptosis in septic liver and lung injuries, where GPX4 expression changed in parallel with YAP1 (Yuan et al. 2022; Zhang et al. 2022). Furthermore, YAP1 has been reported as a transcription factor that influenced target gene expression through its transcription activity (Shibata et al. 2020). More importantly, the JASPAR database predicted potential binding sites between YAP1 and GPX4 promoter. Therefore, we speculated that YAP1 may regulate GPX4 expression, thereby affecting ferroptosis in OP.

Based on this background, we put forward our hypothesis that GPX4, regulated by YAP1, suppresses ferroptosis and enhances osteogenic potential of osteoblasts in OP. Through the design of animal experiments and cell experiments, we verified this hypothesis. We believed our findings will further elucidate the underlying pathological mechanism of OP and provide more targeted therapeutic genes for OP treatment.

## Methods

### Collection of the clinical specimens

In this study, a total of 56 samples, which were respectively obtained from OP patients (28 cases) and healthy volunteers (28 cases). All participants were recruited in The Third Xiangya Hospital of Central South University. Of note, healthy volunteers and post-menopausal OP patients were all women who suffered from hip fractures and underwent surgical treatment. The inclusion criteria of OP patients were: (1) postmenopausal for ≥ 1 year; (2) voluntarily signed the informed consent form before the start of the study. The exclusion criteria of OP patients included: (1) presence of diseases that may significantly interfere with bone metabolism, such as thyroid diseases, diabetes, malignant tumors, kidney diseases, or ankylosing spondylitis; (2) previous treatment with anti-osteoporotic drugs or hormone therapy (previous use of vitamin D and/or calcium supplements is allowed), including estrogen or glucocorticoids, etc. First, we collected some information of participants, including age, BMI, smoking, and drink, which were listed in Supplementary Table 1. Subsequently, the blood and femur were collected for follow-up experiments. The experimental procedures were approved by the Ethics Committee of The Third Xiangya Hospital of Central South University. Informed consents were signed by all participants.

### Reverse transcription quantitative polymerase chain reaction (RT-qPCR)

To evaluate relative expression of key genes, RT-qPCR was conducted. Firstly, total RNA was acquired from clinical samples, mice and MC3T3-E1 cells using TRIzol reagent (Beyotime). Afterwords, by means of Script Reverse Transcription Reagent Kit (TaKaRa, China), cDNA was synthesized. SYBR Premix Ex Taq II Kit (TaKaRa) was used for qPCR process. The primer sequences (Sangon, China) applied in this experiment were exhibited in Table 1. After a standard protocol in a QuantStudio™ 7 Flex Real-Time PCR System (Thermo Fisher Scientific), using 2^−ΔΔCt^ formula, the relative expression was calcuated. β-actin served as reference gene.

**Table 1:** Primer sequences for RT-qPCR

Gene	Forward (5’−3’)	Reverse (5’−3’)
GPX4	CTGCTCTTCCAGAGGTCCTG	GAGGTGTCCACCAGAGAAGC
FTH	CCATCAACCGCCAGATCAAC	CGCCATACTCCAGGAGGAAC
SLC7A11	GTCATCGGATCAGGCATCTT	CATAGGACAGGGCTCCAAAA
NCOA4	GGGACCGGAGCCTTTCGT	TTCATTCTGCCTACTGTTCCAGC
RUNX2	TTCTCCAACCCACGAATGCAC	CAGGTACGTGTGGTAGTGAGT
COL1A1	TCTAGACATGTTCAGCTTTGTGGAC	TCTGTACGCAGGTGATTGGTG
β-actin	GACATGGAGAAGATCTGGCA	GGTCTTTACGGATGTCAACG

### Western blot

Clinical samples, mice and MC3T3-E1 cells serves as experiments subjects and proteins were obtained using RIPA lysis buffer (Beyotime, China). Then, concentrations of protein in different groups were determined by means of bicinchoninic acid (BCA) method. Equal proteins underwent separation through sodium dodecyl sulfate-polyacrylamide gel electrophoresis (SDS-PAGE) and then transferred onto polyvinylidene fluoride (PVDF) membranes, which were blocked by skimmed milk (5%). Afterwards, primary antibodies were used for incubation of PVDF membranes overnight at 4˚C. After three times shaking cleaning with TBST solution, PVDF membranes continued to be incubated by horseradish peroxidase (HRP)-conjugated secondary antibody (Beyotime) for 1 h. Enhanced chemiluminescence (ECL) kit (Beyotime) was used to visualize the protein bands. The densitometry analysis was evaluated by using ImageJ. The information of antibodies used in this experiment were listed below: GPX4 (ab125066, 1:5000), COL1A1 (PA5-29569, 1:2000), RUNX2 (PA5-86506, 1:1000), FTH (ab75973, 1:2000), SLC7A11 (ab307601, 1:1000), NCOA4 (ab314553, 1:1000), YAP1 (PA1-46189, 1:1000), *p*-YAP1 (PA5-17481, 1:1000), and β-actin (MA1-140, 1:10000). Of these, antibodies including GPX4, FTH and SLC7A11 were purchased from Abcam and the rest were purchased from Thermo Fisher Scientific.

### Immunohistochemistry (IHC)

Femur tissues of participants and mice collected and underwent fixation with 4% paraformaldehyde (PFA) and embedding with paraffin. Then, 5 μm thick sections were obtained using pathological microtome. After sections were repaired the antigen and blocked with 1% bovine serum albumin (BSA), antibodies against GPX4 (Abcam), COL1A1 (Thermo Fisher Scientific) and RUNX2 (Thermo Fisher Scientific), FTH (Abcam), SLC7A11 (Abcam), NCOA4 (Abcam) were added into sections and reacted with the corresponding proteins overnight at 4 ℃. Subsequently, HRP-labelled antibody was used for sections and then counterstained with diaminobenzidine (DAB). Finally, the images were acquired using with an optical microscope (Japan).

### Bone densitometry

To detect bone mineral density (BMD) at the lumbar spine of participants including OP patients and heathly volunteers, dual-energy X-ray absorptiometry (DXA) was used for this detection.

### Ovariectomized (OVX) mice model

To evaluate how GPX4 expression affects OP progression, female C57BL/6J mice (8 w), some of which were subjected to GPX4 knockout, which was achieved by CRISPR/Cas9-mediated conditional gene knockout. All mice were constructed by Gempharmatech (Jiangsu, China). In this experiment, mice with/without GPX4 knockout were grouped into WT-Sham, WT-OVX, KO-Sham, KO-OVX and KO-OVX + Ferrostatin-1 (Fer-1; MedChemExpress, USA). Note that the number of mice in each group was 9. Another thing that needed to be noted that WT-Sham represented wild-type C57BL/6J mice; WT-OVX represented wild-type C57BL/6J mice receiving OVX surgery; KO-Sham represented C57BL/6J mice with GPX4 knockout. KO-OVX referred to C57BL/6J mice with GPX4 knockout which inflicted OVX surgery. The treatment process of mice in Sham group was as follows: mice received a surgery which preserved the bilateral ovaries and removed nearby adipose tissue. For mice in OVX group, mice were removed the bilateral ovaries. Fer-1 was administered at a concentration of 1 mg/kg via intraperitoneal injection every other day after OVX. 8 w of surgery later, all mice were sacrificed and biological samples were taken for this study. Animal experiments were approved by the ethics committee of The Third Xiangya Hospital of Central South University.

### Hematoxylin and eosin (HE) staining

Pre-treatment of HE staining were consistent with IHC experiment. After 5 μm thick sections were obtained, sections were dehydrated in ethanol. Afterwards, hematoxylin and eosin were in turn used to dye sections. Finally, images of the stained sections were obtained using an optical microscope with a camera (Olympus, Japan).

### Micro-computed tomography (Micro-CT) analysis

To acquire photographs of bone microstructure and some parameters for OP evaluation, a micro-CT device (Bruker, USA) was used in this study. In brief, the collected femur tissues of mice were fixed with 4% PFA. Referring to a prior study (Zhang et al. 2023), the fixed femur tissues were scanned through micro-CT device. the Main region was chosen 1 ​mm above the distal growth plate. The obtained data was 3D-reconstructed using NRecon Reconstruction Software v1.7.1.6 (Micro Photonics Inc., USA) and analyzed by CTAn Software v1.17.9.0 (Blue Scientific Ltd., UK) and CTvox Software v3.3.0.0 (Blue Scientific Ltd.). The values of parameters including BMD, bone volume fraction (BV/TV), trabecular number (Tb.N.), trabecular thickness (Tb.Th.), trabecular separation (Tb.Sp) and bone surface (BS/BV) were obtained to evaluate trabecular microarchitecture and OP progression.

### Cell culture and treatment

The mouse osteoblastic cell line MC3T3-E1 was obtained from Cell Bank of the Chinese Academy of Sciences (Shanghai, China). All cells were cultured in α -MEM medium (Thermo Fisher Scientific, USA) supplemented with 10% fetal bovine serum (FBS; Thermo Fisher Scientific), 100U/ml penicillin at 37 °C in a humidified atmosphere of 5% CO_2_. To induce osteogenic differentiation, MC3T3-E1 cells were seeded into 6-well plates and cultured in osteogenic differentiation medium following a previous study (Yang et al. 2021a, b).

For erastin treatment, 25 µM erastin (Selleck Chemicals, USA) was added into MC3T3-E1 cells for 12 h incubation. For LAT1-IN-1 treatment, 30 mM LAT1-IN-1 (MedChemExpress, USA) was added into MC3T3-E1 cells for 24 h incubation.

### Cell transfection

Overexpressing plasmid of GPX4 (ov-GPX4) or YAP1 (ov-YAP1) and short hairpin targeting GPX4 (sh-GPX4) along with correspondent controls (ov-NC, sh-NC) were purchased from GenePharma (China). MC3T3-E1 cells were implanted onto 6-well plates and incubated overnight. MC3T3-E1 cells were transfected with plasmids for 48 h using Lipofectamine™ 3000 (Invitrogen, USA) following the instructions. After induction for 14 d, MC3T3-E1 cells were subjected to other substances’ intervention.

### Immunofluorescence (IF) assay

Tissue samples and MC3T3-E1 cells were collected. The experimental subjects were fixed with 4% PFA and then were permeabilized with 0.5% Triton X-100 solution. Subsequently, samples were blocked with 5% BSA. The primary antibodies against GPX4 (Abcam), RUNX2 (Abcam), YAP1 (Thermo Fisher Scientific) were used for incubation of samples overnight at 4 °C. Besides, samples were ubcubated with secondary antibodies. After that, 4′,6-Diamidino-2-Phenylindole (DAPI) was applied for staining cell nucleus. The images were pictured using a fluorescence microscope.

### Transmission electron microscopy (TEM)

MC3T3-E1 cells were processed according to detailed steps in a previous study(Ke et al. 2021). Finally, the ultramicroscopic images of mitochondria were observed under a TEM (FEI, USA).

### Detection of malondialdehyde (MDA), glutathione (GSH), reactive oxygen species (ROS) and Fe^2+^ levels

The MDA contents in mouse serum and femur and MC3T3-E1 cells were measured using an MDA assay kit (Beyotime, China) according to the manufacturer’s instructions. The GSH contents in mouse serum and femur and MC3T3-E1 cells were measured using a GSH assay kit (Beyotime) following the manufacturer’s protocol. The ROS levels in MC3T3-E1 cells were evaluated using a ROS detection kit (Beyotime). Additionally, the Fe^2+^ level in MC3T3-E1 cells was measured using an iron Assay kits (Solarbio).

### Lipid peroxidation (LPO) assay

The C11-BODIPY 581/591 kit (Thermo Fisher Scientific) was used to detect LPO in MC3T3-E1 cells according to the manufacturer’s instructions. Shortly, the reagent was added to 96-well plates, and MC3T3-E1 cells were incubated for 30 min, followed by washing with PBS. Cell nuclei were stained with DAPI (Thermo Fisher Scientific). Subsequently, observations and analyses were performed using a fluorescence microscope (Leica, Germany) and Fiji software.

### Alkaline phosphatase (ALP) staining and alizarin red S (ARS) staining

ALP staining and ARS staining were conducted to evaluate osteogenic capability. In short, collected MC3T3-E1 cells were subjected to fixation with 4% PFA. For ALP staining, MC3T3-E1 cells were incubated with ALP staining solution (Solarbio, China) for 20 min. For ARS staining, MC3T3-E1 cells were incubated with 2% Alizarin red S reagent (sigma) for 15 min. Stained cells were imaged using a microscope (Nikon, Japan).

### Chromatin immunoprecipitation (ChIP) assay

The ChIP assay was conducted with the Simple ChIP^®^ Enzymatic Chromatin IP Kit (Cell Signaling Technology, USA). Briefly, after fixation with formaldehyde, cells were quenched with glycine. Chromatin was digested enzymatically and subjected to immunoprecipitation using a YAP1-specific primary antibody (PA1-46189, Thermo Fisher Scientific), with incubation carried out overnight at 4 °C. Antibody-chromatin complexes were isolated using protein A/G magnetic beads. After reversing the crosslinks, the immunoprecipitated DNA was purified and quantified via qPCR.

### Dual-luciferase reporter gene assay

To determine the binding sites of YAP1 on the GPX4 promoter, potential binding sequences were predicted using the JASPAR database (https://jaspar.elixir.no/). These sites were subsequently mutated through site-directed mutagenesis. Both the wild-type (WT) and mutant (MUT) GPX4 promoter fragments were cloned into pGL3-Basic luciferase reporter vectors. For luciferase reporter assays, MC3T3-E1 cells were co-transfected with either an overexpression control plasmid (ov-NC) or a YAP1 overexpression plasmid (ov-YAP1), together with the respective reporter constructs, using Lipofectamine™ 3000. Following 48 h of incubation at 37 °C under 5% CO₂, luciferase activity was quantified with the Dual-Glo^®^ Luciferase Assay System (Promega, USA).

### Statistical analysis

Statistical analyses were performed using GraphPad Prism 9. All data in this study were obtained from 3 independent experiments and were presented as mean ± standard deviation (SD). For statistical comparisons, Student’s t-test was used for analyzing differences between two groups and one-way analysis of variance (ANOVA) was applied for comparisons among more than two groups. Pearson correlation analysis was used to assess the relationships between genes. A *p*-value < 0.05 was considered statistically significant.

## Results

### GPX4 expression was decreased in clinical samples of OP and positively correlated with BMD

**Fig. 1:**
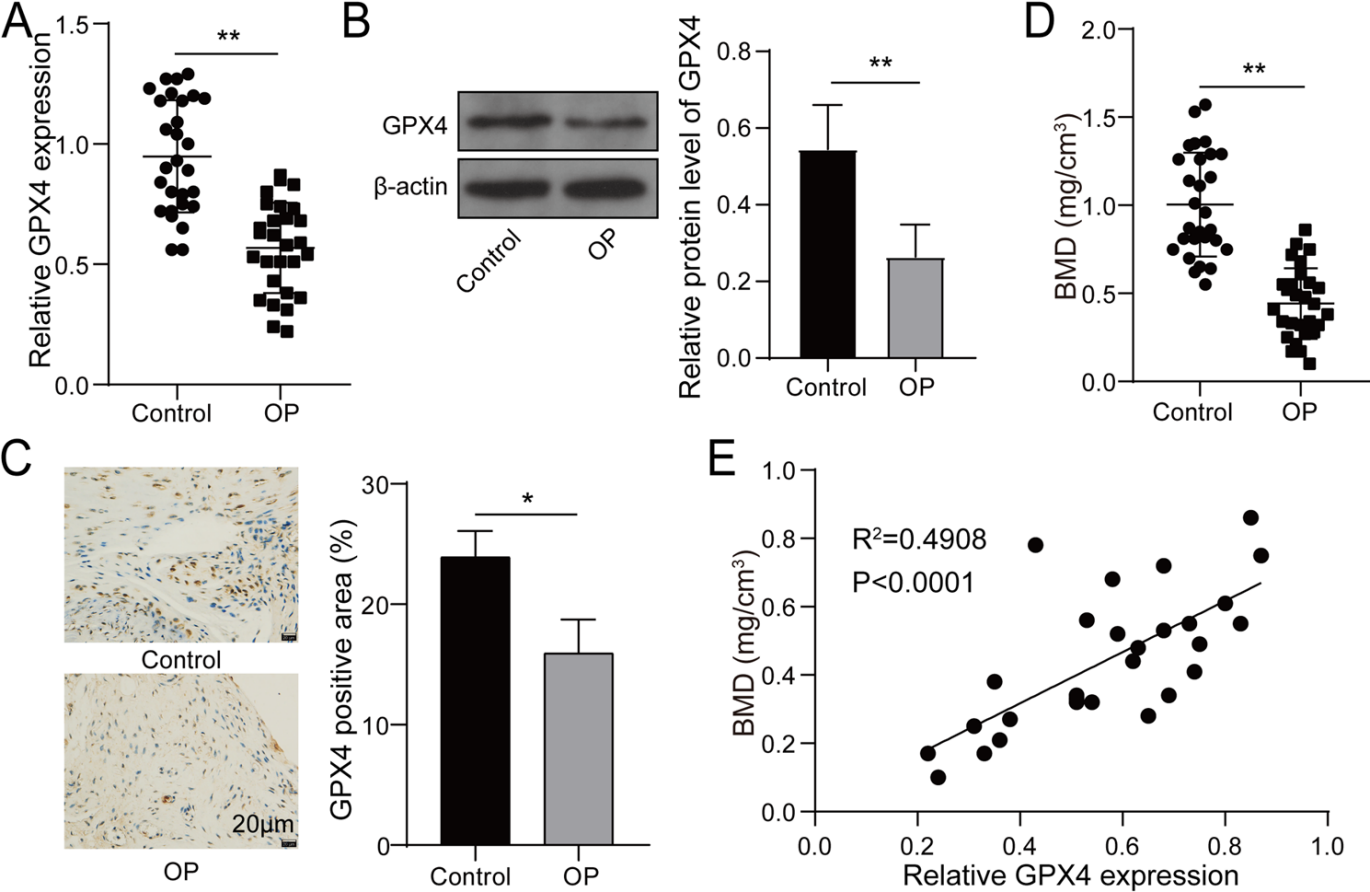
GPX4 expression was decreased in clinical samples of OP and positively correlated with BMD. (**A**) GPX4 expression in blood from female patients with/without OP was examined using RT-qPCR (*n* = 28). (**B**-**C**) GPX4 expression in femur tissues from female patients with/without OP was determined by means of western blot (*n* = 28) and IHC (*n* = 3). (**D**) BMD of lumbar vertebra of participants was obtained using DXA (*n* = 28). (**E**) The relationship between GPX4 expression and BMD in OP patients was analyzed using Pearson correlation analysis. *P < 0.05, **P < 0.01

GPX4 has been reported to participate in osteoclast formation in OP (Deng et al. 2023). Here, to investigate GPX4 expression in OP, tissue samples, including serum and femur tissues, were collected from female patients with/without OP. First, we gathered baseline data, including age, BMI, smoking, and drink, from both healthy volunteers and patients with OP. Statistical analysis revealed that there were no significant differences between the two groups in terms of these four baseline characteristics (Supplementary Table 1). Besides, RT-qPCR determined that GPX4 expression in serum of OP patients was notably lower than that in normal controls **(**Fig. 1A**)**. Likewise, in femur tissues, western blot and IHC detection also revealed that GPX4 expression was apparently decreased in OP patients **(**Fig. 1B-C**)**. In addition, we found that the lumbar vertebra BMD of OP patients was lower compared with normal participants **(**Fig. 1D**)**. Further analysis revealed a positive relationship between GPX4 expression and BMD in OP patients **(**Fig. 1E**)**. Collectively, GPX4 is probably associated with OP and may play a regulatory role in OP progression.

### GPX4 knockout aggravated OP in mice

**Fig. 2:**
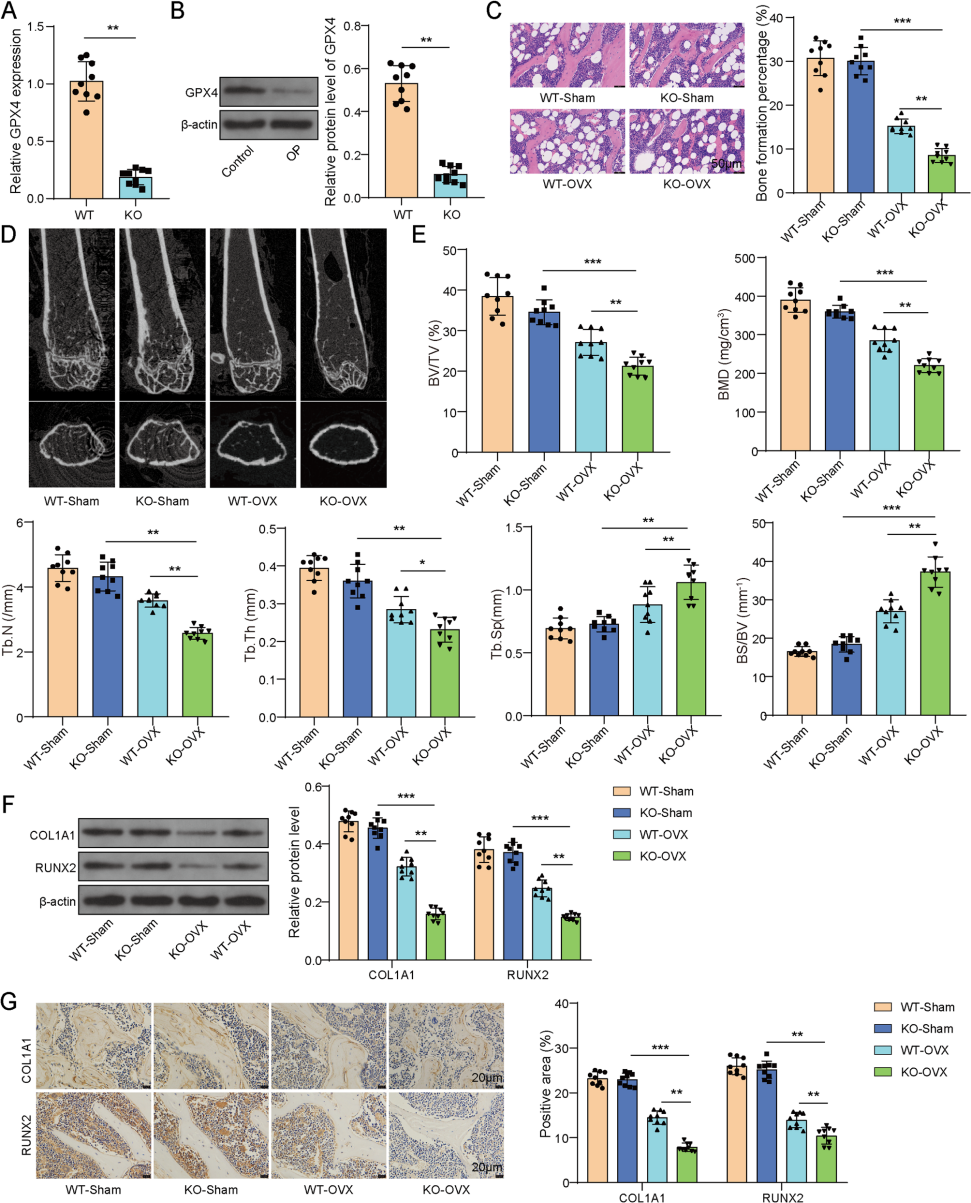
GPX4 knockout aggravated OP in mice. The mice were randomly divided into four experimental groups with different treatments: (1) WT-Sham group (wild-type mice receiving sham surgery); (2) KO-Sham group (GPX4-knockout mice receiving sham surgery); (3) WT-OVX group (wild-type mice subjected to OVX modeling); and (4) KO-OVX group (GPX4-knockout mice subjected to OVX modeling). (**A**-**B**) GPX4 expression in GPX4 knockout mice and WT mice was acquired by RT-qPCR and western blot. (C-G) GPX4 knockout mice and WT mice received OVX surgery to mimic OP. (**C**) The morphology of femur was examined by HE staining. (**D**) The microstructure of femur were obtained using micro-CT. (**E**) The parameters including BV/TV, BMD, Tb.N, Tb.Th, Tb.Sp and BS/BV were analyzed using micro-CT. (**F**-**G**) COL1A1 and RUNX2 expression in femur of mice was determined using western blot and IHC. *n* = 9. *P < 0.05, **P < 0.01, ***P < 0.001

To investigate the effect of GPX4 on OP, GPX4 knockout mice were purchased and then some of the GPX4 knockout mice and WT mice underwent OVX surgery to induce OP models. Firstly, as presented in Fig. 2A-B, GPX4 expression in GPX4 knockout mice was greatly declined compared to WT mice, indicating successful GPX4 knockout. Subsequently, WT mice were randomly grouped into WT- Sham and WT-OVX and GPX4 knockout mice were randomly divided into KO-Sham and KO-OVX. HE staining of femoral morphology showed that in WT-Sham and KO-Sham groups, bone trabeculae were neatly arranged with good continuity and intact structure. However, in WT-OVX group, bone trabeculae were fewer, disordered, unevenly arranged, and poorly connected. These changes were further exacerbated in KO-OVX group **(**Fig. 2C**)**. Micro-CT was used to obtain radiographic images of all mice and relevant parameters were compared between groups. Compared to WT-Sham group, BV/TV, BMD, Tb.N and Tb.Th of mice were dramatically declined in WT-OVX group, while Tb.Sp and BS/BV was observably increased. GPX4 knockout further reinforced these parameter changes **(**Fig. 2D-E**)**. Additionally, western blot and IHC analyses showed that the expression of osteogenic markers including COL1A1 and RUNX2 was lower in WT-OVX mice and their expression was further declined in KO-OVX mice **(**Fig. 2F-G**)**. Taken together, GPX4 knockout exacerbated bone loss and impaired osteogenic potential, thereby promoting OP progression in mice.

### GPX4 knockout promoted ferroptosis in mice with OP

**Fig. 3:**
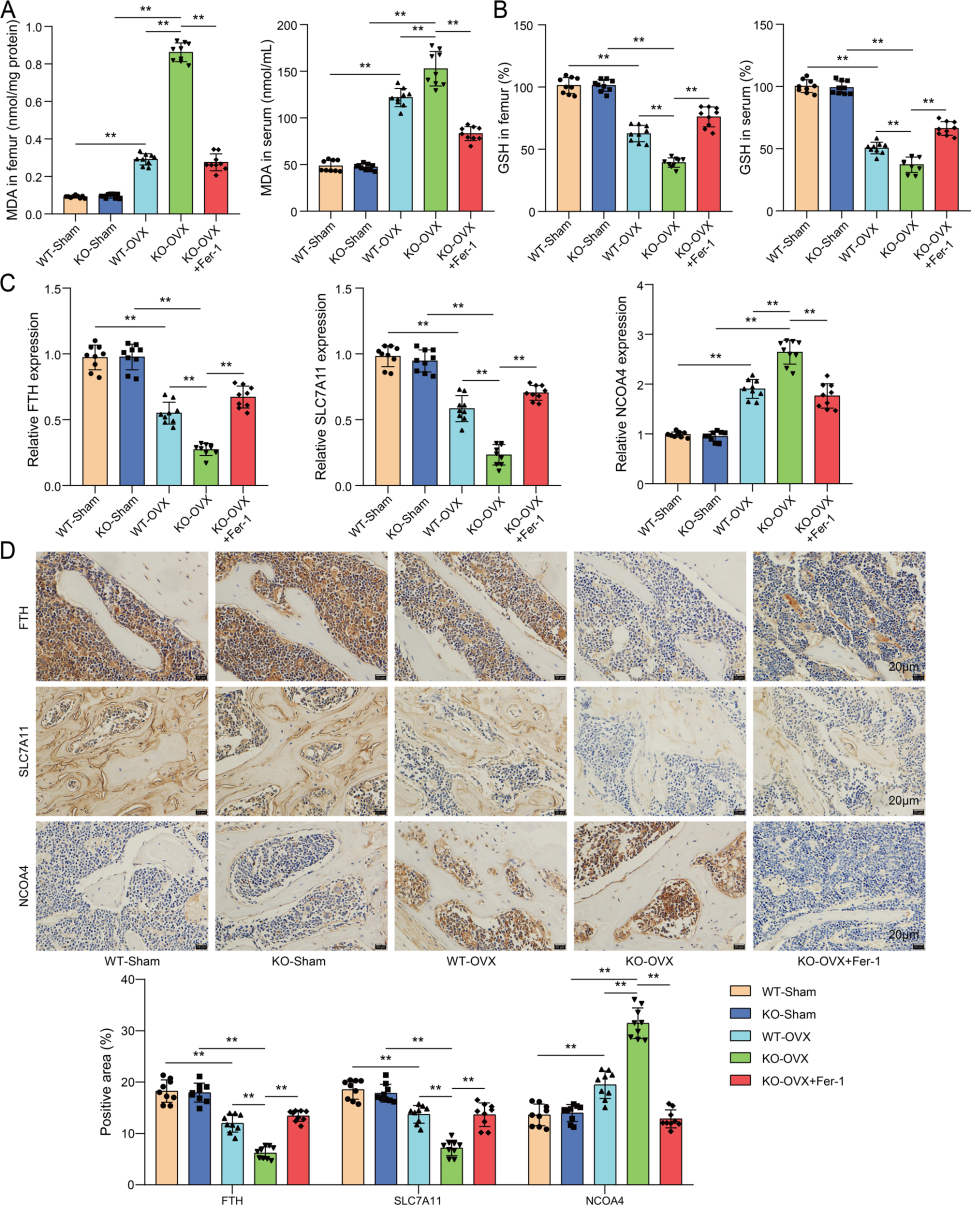
GPX4 knockout promoted ferroptosis in mice with OP. GPX4 knockout mice and WT mice received OVX to simulate OP and GPX4-knockout OP mice were subjected to Fer-1 administration. (**A**-**B**) MDA and GSH levels in serum and femur of mice were examined using commercial kits. (**C**-**D**) FTH, SLC7A11 and NCOA4 expression in femur of mice was evaluated using RT-qPCR and IHC. *n* = 9. **P < 0.01

As previous documented, suppressing ferroptosis could alleviate OP progression(Yan et al. 2022). To probe whether ferroptosis is implicated in OP progression and whether GPX4 affects ferroptosis in OP mice, ferroptosis-related indicators were detected in samples from different mouse groups (WT-Sham, WT-OVX, KO-Sham, KO-OVX, KO-OVX + Fer-1). Notably, to clarify the role of ferroptosis in GPX4 knockout OP mice, we intervened in the mice using the ferroptosis inhibitor Fer-1. As shown in Fig. 3A, the levels of MDA in serum and femur of WT-OVX mice were evidently enhanced and GPX4 knockout elevated MDA levels. However, Fer-1 treatment reversed GPX4 knockout-mediated promotive effects on MDA levels. Conversely, GSH levels were decreased in serum and femur of WT-OVX mice and these changes were exacerbated by GPX4 knockout. As expected, the suppressive effects on GSH levels mediated by GPX4 knockout were abolished by Fer-1 treatment **(**Fig. 3B**)**. Furthermore, RT-qPCR and IHC demonstrated lower expression of FTH and SLC7A11 and higher expression of NCOA4 in femur of WT-OVX mice and these trends were enhanced by GPX4 knockout. Besides, GPX4 knockout-mediated decrease in FTH and SLC7A11 and increase in NCOA4 was compromised by Fer-1 treatment **(**Fig. 3C-D**)**. Collectively, GPX4 knockout promoted ferroptosis in OP mice.

### GPX4 overexpression suppressed ferroptosis in osteoblasts

**Fig. 4:**
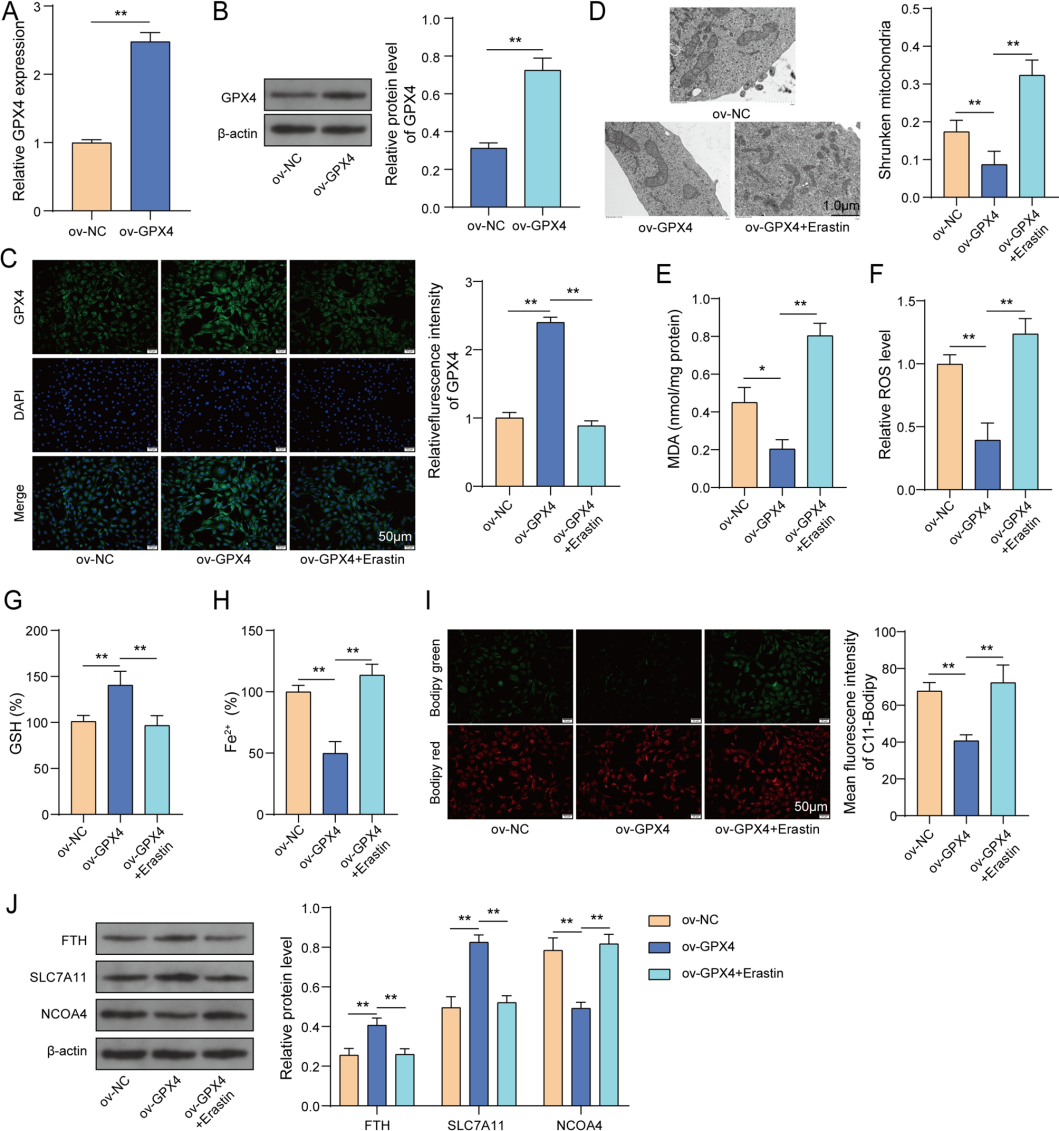
GPX4 overexpression suppressed ferroptosis in osteoblasts. MC3T3-E1 cells were treated with ov-NC, ov-GPX4, ov-GPX4 + Erastin. (**A**-**C**) GPX4 expression was determined using RT-qPCR, western blot and IF assay, respectively. (**D**) Ultrastructural changes of mitochondria were observed using TEM. (**E**-**H**) MDA, ROS and Fe^2+^ and GSH levels in cells were examined using commercial kits. (**I**) Lipid peroxidation was detected using Bodipy-C11 fluorescent probe. (**J**) FTH, SLC7A11 and NCOA4 expression was examined using western blot. *n* = 3. *P < 0.05, **P < 0.01

Here, we continued to examine the influence of GPX4 on ferroptosis in osteoblasts. Mouse precursor osteoblasts (MC3T3-E1 cells) were used and transfected with ov-NC or ov-GPX4. As indicated in Fig. 4A-B, the mRNA and protein level of GPX4 were elevated significantly in MC3T3-E1 cells transfected ov-GPX4. Then, the transfected MC3T3-E1 cells induced by osteogenic differentiation medium or treat with Erastin (a ferroptosis agonist). The detailed groups were as follows: ov-NC, ov-GPX4, ov-GPX4 + Erastin. As indicated in Fig. 4C, ov-GPX4 transfection increased GPX4 expression in MC3T3-E1 cells. However, Erastin compromised GPX4 overexpression-mediated elevation of GPX4 expression. Then, the ultrastructural changes of mitochondria were observed by TEM. GPX4 overexpression decreased percentage of shrunken mitochondria, while Erastin administration significantly reversed the effect of GPX4 overexpression, displaying morphological features of ferroptosis, such as shrunken mitochondria, thicker membranes, reduced or absent mitochondrial crists, and a significant increase in the percentage of shrunken mitochondria **(**Fig. 4D**)**. Furthermore, GPX4 overexpression-mediated reduction of MDA, ROS and Fe^2+^ levels and enhancement of GSH level were offset by Erastin treatment **(**Fig. 4E-H**)**. As presented in Fig. 4I, Bodipy-C11 fluorescent probe indicated that GPX4 overexpression resulted in less lipid peroxidation; however, Erastin treatment reversed this effect of GPX4 overexpression. Besides, Erastin treatment reversed GPX4 overexpression-mediated upregulation of FTH, SLC7A11 and downregulation of NCOA4 in MC3T3-E1 cells **(**Fig. 4J**)**. Taken together, GPX4 overexpression could inhibit ferroptosis in osteoblasts.

### GPX4 overexpression promoted osteogenic capabilities of osteoblasts via inhibiting ferroptosis

**Fig. 5:**
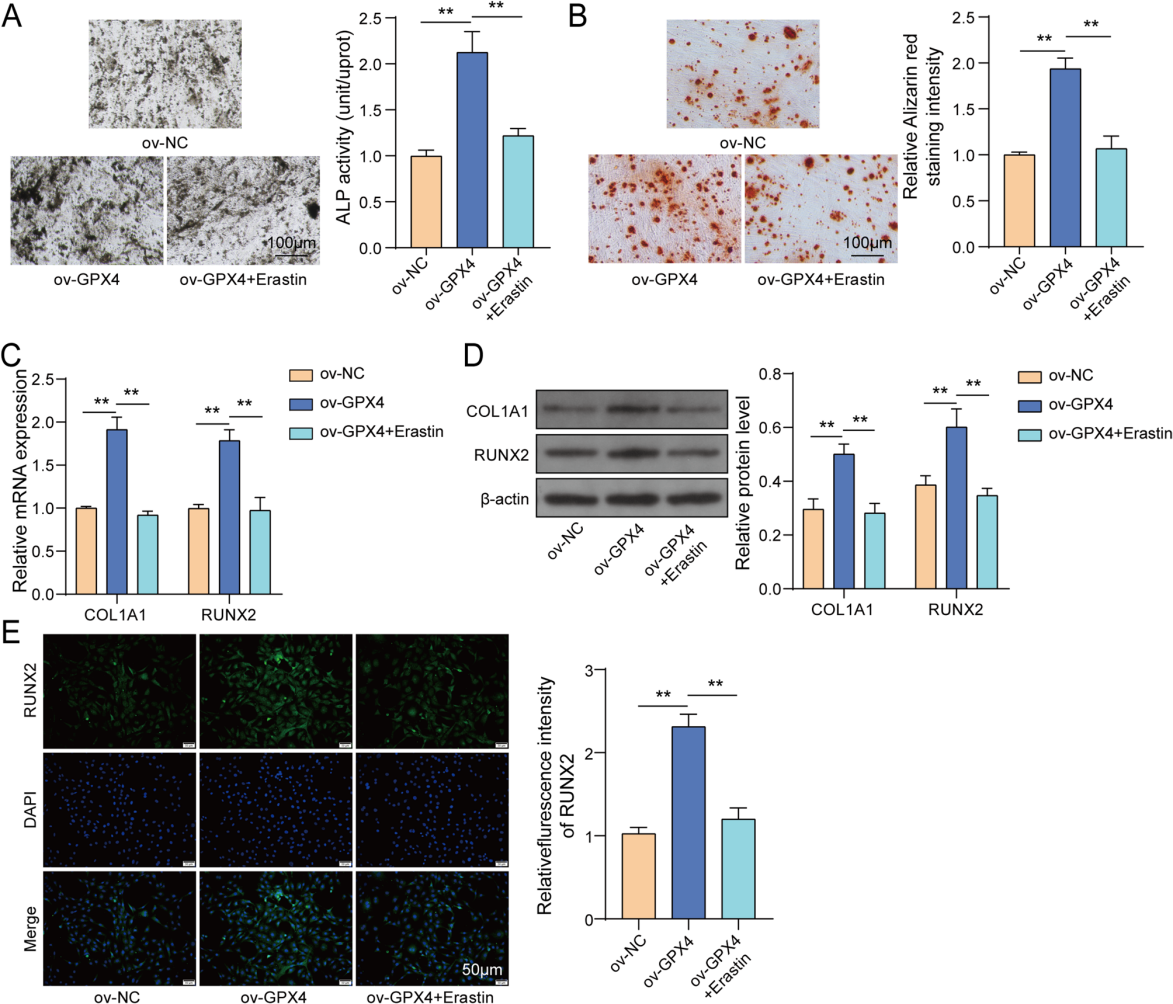
GPX4 overexpression promoted osteogenic capabilities of osteoblasts via inhibiting ferroptosis. MC3T3-E1 cells were treated with ov-NC, ov-GPX4, ov-GPX4 + Erastin. (**A**) ALP activity was measured using ALP staining. (**B**) Calcified nodules were examined using ARS staining. (**C**-**D**) COL1A1 and RUNX2 expression was determined using RT-qPCR and western blot, respectively. (**E**) RUNX2 expression was determined by IF assay. *n* = 3. **P < 0.01

To investigate how GPX4 influences osteogenic differentiation of osteoblasts, we evaluated the effects of ov-GPX4 transfection and Erastin treatment on osteogenic capacity. ALP staining and ARS staining showed that GPX4 overexpression significantly increased ALP activity and the number of calcified nodules. However, Erastin reversed GPX4 overexpression-mediated enhancement of ALP activity and calcified nodules **(**Fig. 5A-B**)**. Moreover, GPX4 overexpression enhanced COL1A1 and RUNX2 expression in MC3T3-E1 cells, whereas Erastin compromised the impacts of GPX4 overexpression on COL1A1 and RUNX2 expression **(**Fig. 5C-E**)**. To sum up, GPX4 overexpression promoted osteogenic differentiation of osteoblasts by suppressing ferroptosis.

### YAP1 regulated positively GPX4 expression in osteoblasts

**Fig. 6:**
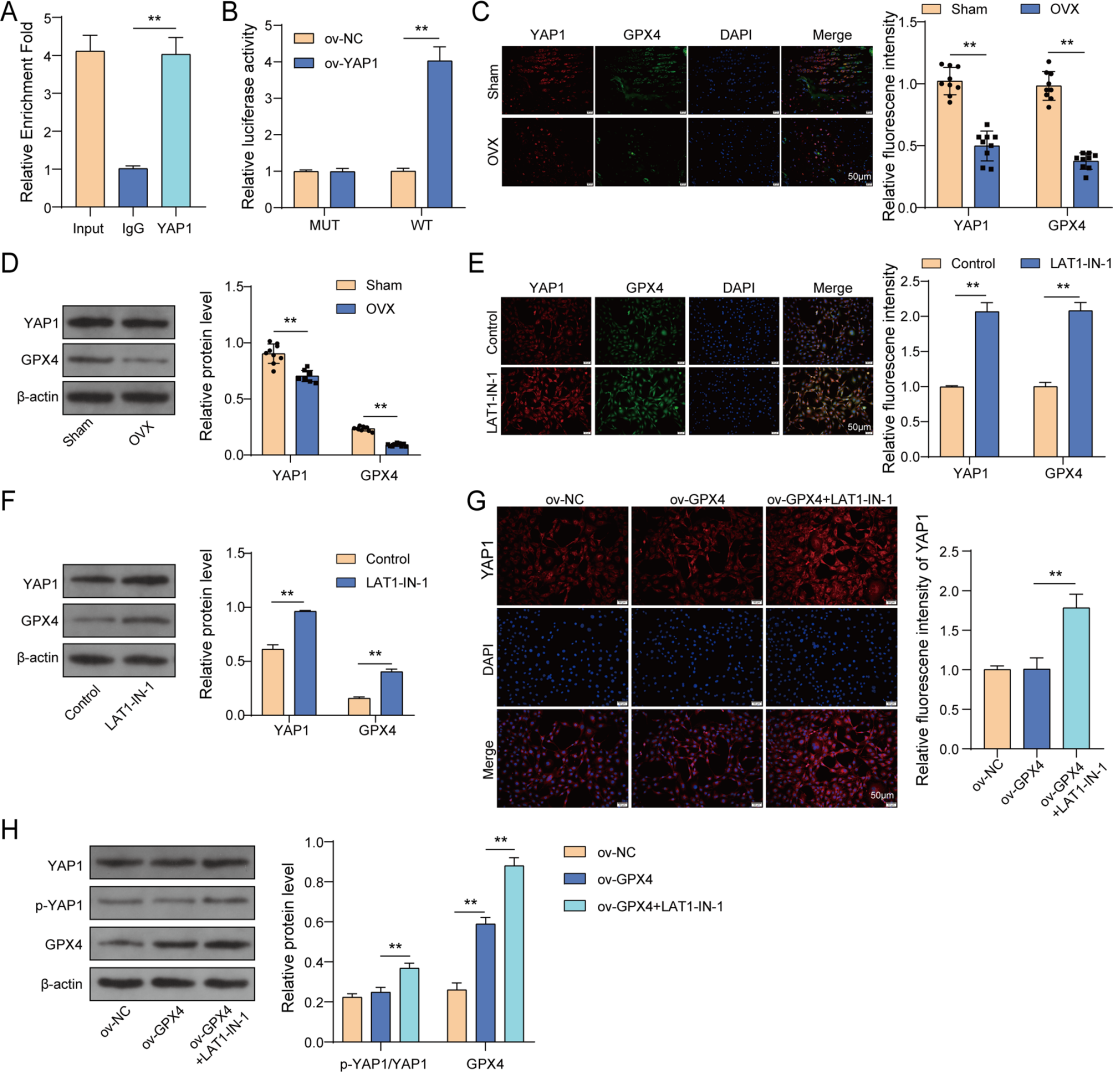
YAP1 regulated positively GPX4 expression in osteoblasts. (**A**-**B**) The interaction between YAP1 and GPX4 promoter was validated using ChIP and dual-luciferase reporter gene assay. (**C**) YAP1 and GPX4 expression in femur of OVX-induced mice was measured using IF assay. (**D**) YAP1 and GPX4 expression in femur of OVX-induced mice was determined using western blot. (**E**-**F**) MC3T3-E1 cells received LAT1-IN-1 treatment. YAP1 and GPX4 expression was measured using IF (E) and western blot assay (**F**). (**G**-**H**) Ov-GPX4 transfected MC3T3-E1 cells received LAT1-IN-1 treatment. (**G**) YAP1 expression was measured using IF assay. (**H**) GPX4, YAP1 and *p*-YAP1 expression was determined using western blot. *n* = 3. *P < 0.05, **P < 0.01

YAP1, a terminal effector in the Hippo pathway, was implicated in OP (Zhong et al. 2021). Besides, YAP1 functioned as a transcription factor to regulate gene expression (Shibata et al. 2020). In addition, studies have suggested that YAP1 could inhibit ferroptosis, accompanied by increased GPX4 expression in septic liver injury (Wang et al. 2022). Thus, we hypothesized that YAP1 might regulate the transcriptional activity of GPX4. To validate the interaction between YAP1 and the GPX4 promoter, we first used the JASPAR database to predict potential binding sites between the two (Fig S1A-1B). Then, ChIP assays demonstrated that YAP1 antibody successfully enriched the GPX4 promoter **(**Fig. 6A**)**. Moreover, overexpression of YAP1 enhanced luciferase activity in the GPX4-wt group but had no effect on the GPX4-mut group **(**Fig. 6B**)**. These results indicated a direct interaction between YAP1 and the GPX4 promoter. Besides, IF assay and western blot revealed that, in femur samples of mice, YAP1 and GPX4 expression was both decreased observably in OVX-induced mice **(**Fig. 6C-D**)**. To investigate whether YAP1 is responsible for regulating GPX4 expression in OP, MC3T3-E1 cells were treated with LAT1-IN-1 (a YAP activator), and the changes of GPX4 expression were evaluated. Using IF and western blot assays, we found that LAT1-IN-1 treatment could apparently enhanced YAP1 and GPX4 expression in MC3T3-E1 cells **(**Fig. 6E-F**)**. Subsequently, MC3T3-E1 cells was subjected to ov-GPX4 transfection with the following groups: ov-NC, ov-GPX4, ov-GPX4 + LAT1-IN-1. Results from IF assay and western blot assay exhibited that ov-GPX4 transfection did not influence YAP1 expression, whereas LAT1-IN-1 treatment largely increased YAP1 expression. In addition, we observed that ov-GPX4 transfection had no effect on YAP1 and p-YAP1 expression, while LAT1-IN-1 treatment increased their expression in MC3T3-E1 cells **(**Fig. 6G-H**)**. Taken together, YAP1 could increase GPX4 expression in MC3T3-E1 cells through activating its transcriptional activity.

### YAP1 suppressed ferroptosis and enhanced osteogenic capability of osteoblasts via elevating GPX4 expression

**Fig. 7:**
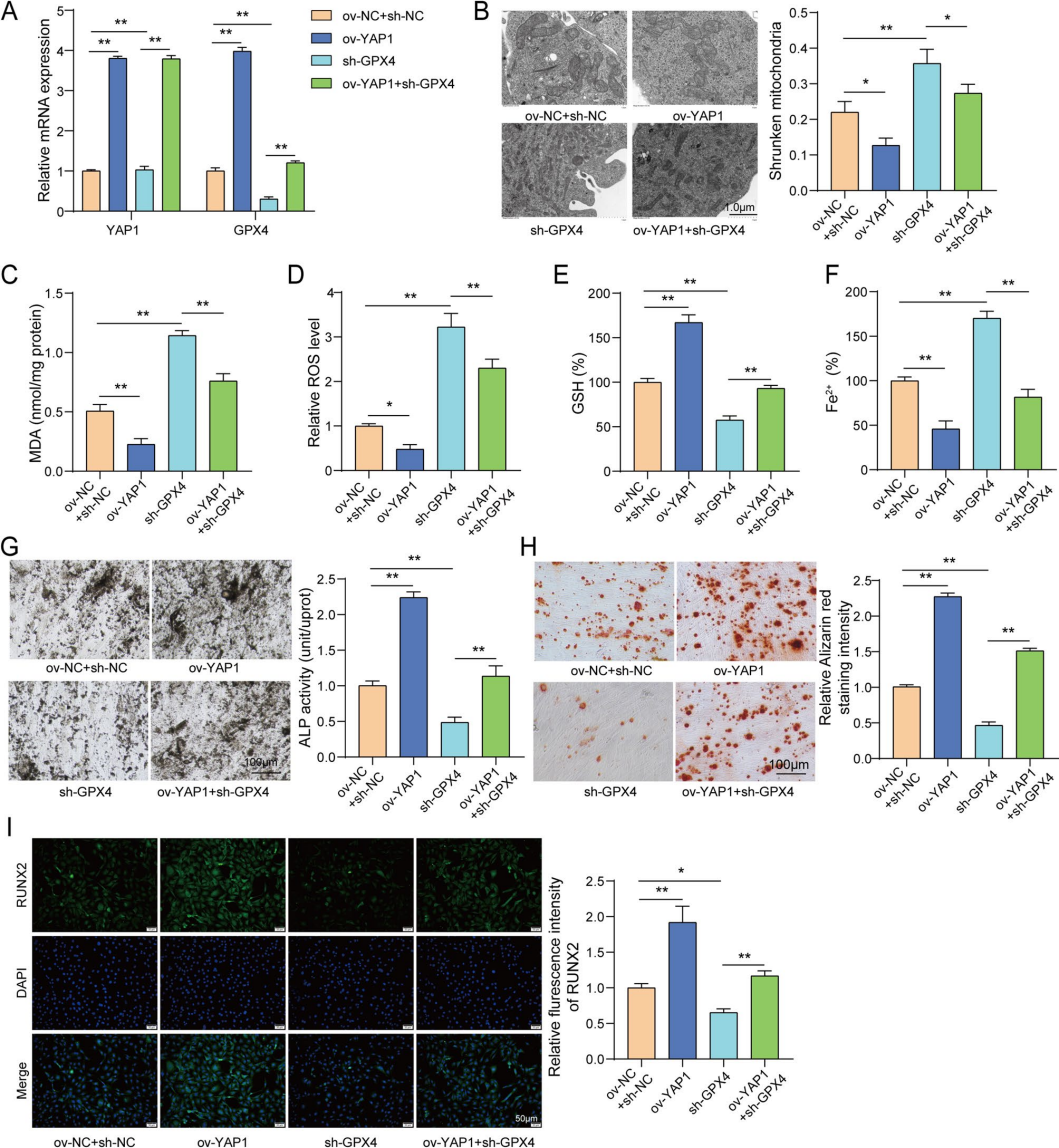
YAP1 suppressed ferroptosis and enhanced osteogenic capability of osteoblasts via elevating GPX4 expression. MC3T3-E1 cells were transfected with ov-YAP1 or/and sh-GPX4. (**A**) YAP1 and GPX4 expression was determined using RT-qPCR. (**B**) Ultrastructural changes of mitochondria were observed using TEM. (**C**-**F**) MDA, ROS, GSH and Fe^2+^ levels in cells were examined using commercial kits. (**G**) ALP activity was measured using ALP staining. (**H**) Calcified nodules were examined using ARS staining. (**I**) RUNX2 expression was determined by IF assay. *n* = 3. *P < 0.05, **P < 0.01

To explore the role of YAP1/GPX4 axis in OP, MC3T3-E1 cells were transfected with ov-YAP1 or/and sh-GPX4. Firstly, YAP1 overexpression resulted in increased YAP1 and GPX4 expression in MC3T3-E1 cells. GPX4 knockdown only decreased GPX4 expression, and this effect was reversed by YAP1 overexpression **(**Fig. 7A**)**. YAP1 overexpression decreased percentage of shrunken mitochondria, while GPX4 knockdown observably increased percentage of shrunken mitochondria. As expected, YAP1 overexpression effectively alleviated GPX4 knockdown-mediated ferroptotic morphological features in MC3T3-E1 cells **(**Fig. 7B**)**. In addition, YAP1 overexpression resulted in reduced levels of MDA, ROS and Fe^2+^, along with increased GSH levels; however, these effects were reversed GPX4 knockdown **(**Fig. 7C-F**)**. Moreover, YAP1 overexpression promoted ALP activity and the formation of calcified nodules in MC3T3-E1 cells while GPX4 knockdown exerted the opposite effects and reversed the functions of YAP1 overexpression **(**Fig. 7G-H**)**. Regarding changes in osteogenic marker, IF assay results showed that the inhibitory effect of sh-GPX4 on RUNX2 expression in MC3T3-E1 cells was neutralized by YAP1 overexpression **(**Fig. 7I**)**. These data revealed that YAP1 suppressed ferroptosis and enhanced osteogenic differentiation of osteoblasts via elevating GPX4 expression.

## Discussion

The onset of OP is closely related to age. With the gradual increase in age, a large number of elderly individuals worldwide are affected by the harm caused by OP, with fractures being the most common complication, which greatly impairs the quality of life of the elderly (Srivastava and Deal 2002). Therefore, exploring the pathogenesis and therapeutic strategies for OP is crucial. Encouragingly, accumulating evidences has indicated that enhanced ferroptosis contributed to OP development, indicating that suppressing ferroptosis could delay OP development (Gao et al. 2022a, b). In this study, we confirmed the presence of enhanced ferroptosis in OVX-induced mice through detecting ferroptosis-related markers. Furthermore, our findings demonstrated that GPX4, which was positively regulated by YAP1, could alleviate ferroptosis in OP mice and promote osteogenic potential of osteoblasts to delay OP progression.

Since the discovery of ferroptosis in 2012, it has emerged as a research hotspot in recent years, with implications for the pathogenesis and progression of various diseases (Jiang et al. 2021). As previously documented, bone-related diseases including OP, osteoarthritis, and osteosarcoma, were associated with dysregulated ferroptosis (Gao et al. 2022a, b). For instance, iron overload-induced ferroptosis in osteoblasts accelerated the progression of OP (Jiang et al. 2022). Jian Hao et al. suggested that qing`e pill could alleviate OP progression through regulating PI3K/AKT pathway to inhibit osteoblast ferroptosis (Hao et al. 2022). In current study, we found altered levels of ferroptosis-related indicators, including MDA, GPX4, GSH, FTH, NCOA4 and SLC7A11 in OVX-induced mice, suggesting enhanced ferroptosis. Notably, accumulating evidence has demonstrated that GPX4 can serve as a marker for evaluating cellular ferroptosis (Xue et al. 2023; Yang et al. 2014). Encouragingly, several studies have indicated that GPX4 was involved in the progression of OP. For example, in D-gal-induced aging mice, MC3T3-E1 cells and primary rat osteoblasts, vitamin D receptor (VDR) activation restricted osteoblastic ferroptosis through elevating GPX4 expression, thereby alleviating age-related OP (Xu et al. 2022). Besides, in high glucose-induced hFob1.19 cells and type 2 diabetic OP rats, acid sphingomyelinase promoted ferroptosis through degrading GPX4, accelerating type 2 diabetic OP (Du et al. 2023). In addition, METTL14 enhanced GPX4 mRNA stability to suppress osteoclast formation, thereby ameliorating OVX-induced OP in mice(Deng et al. 2023). GPX4 was transcriptionally suppressed through DNMT-mediated promoter hypermethylation, which induced osteoblastic ferroptosis and promoted OVX-induced OP (Ruan et al. 2024). To further investigate the role of GPX4 in OP, we designed a series of in vivo and in vitro experiments. Our findings revealed that GPX4 expression was reduced in post-menopausal OP female patients and positively correlated with BMD. Additionally, GPX4 knockout in OVX-induced OP mice resulted in a decreased number of bone trabeculae and inhibited bone formation. Furthermore, GPX4 knockout exacerbated OVX-induced ferroptosis in mice, whereas Fer-1 (a ferroptosis inhibitor) attenuated the promotional effect of GPX4 knockout on ferroptosis. Meanwhile, GPX4 overexpression suppressed ferroptosis and enhanced the osteogenic capacity of osteoblasts, which was reversed by Erastin (a ferroptosis agonist). Taken together, these published findings and our results suggested GPX4 played a crucial role in OP progression through mediating ferroptosis.

YAP1, a key downstream effector of Hippo pathway, was implicated in various biological behaviors, such as cell growth, differentiation, apoptosis and ferroptosis (Halder and Johnson 2011; Li et al. 2022; Wang et al. 2023a, b). For example, Yongxiong Huang et al. revealed that circ_0024097 elevated YAP1 expression through sponging miR-376b-3p, thereby promoting osteogenic differentiation in OP (Huang et al. 2020). Extracellular vesicles derived from bone marrow mesenchymal stem cells elevated YAP1 protein stability, which in turn promoted bone formation and suppressed OP progression in mice (Wang et al. 2023a, b). Ri-Xu Liu et al. demonstrated that Trim21 depletion upregulates YAP1 expression, thereby alleviating bone loss in OP (Liu et al. 2023). Additionally, YAP1 expression, regulated by circ-ITCH/miR-214 axis, promoted osteogenic differentiation in OP (Zhong et al. 2021). More importantly, Y YAP1 ameliorates PM2.5-induced lung injury by upregulating SLC7A11 levels to inhibit ferroptosis (Wang et al. 2023a, b). Based on this evidence, we proposed that YAP1 was closely associated with OP progression, and this association may be linked to the regulation of ferroptosis. In this study, we found that YAP1 expression is decreased in OVX-induced mice, and YAP1 overexpression suppressed ferroptosis and enhanced the osteogenic potential of osteoblasts.

Notably, YAP1 can function as a transcription factor to regulate the expression of target genes. For instance, YAP1 transcriptionally upregulates IL6 to promote STAT3 phosphorylation, thereby enhancing cancer stem cell properties, increasing chemotherapy resistance, and driving lung cancer progression (Shibata et al. 2020). Besides, YAP1 could suppress ferroptosis process and enhanced GPX4 expression to alleviate sepsis-induced acute lung injury in mice(Zhang et al. 2022). Similarly, YAP1 improved septic liver injury through suppressing ferroptosis, which was accompanied by elevation of GPX4 expression (Wang et al. 2022). Thus, YAP1 probably regulates GPX4 expression through its transcriptional activity on GPX4 promoter, thereby affecting ferroptosis in OP. In current study, we first validated the interaction between YAP1 and GPX4 promoter. Then, we confirmed that YAP1 and GPX4 were downregulated in OVX-induced mice. In addition, a YAP1 activator could induce upregulation of GPX4 expression while GPX4 overexpression failed to influence YAP1 expression, indicating that YAP1 was an upstream regulator for GPX4 expression. Importantly, we demonstrated that YAP1 expression suppressed of ferroptosis and promoted osteogenic differentiation of osteoblasts. Furthermore, GPX4 knockdown-mediated promotion of ferroptosis and inhibition of osteogenic differentiation of osteoblasts were partial reversed by YAP1 expression.

## Conclusions

In conclusion, we revealed that GPX4 expression was abnormally downregulated in clinical samples and OVX-induced mice. More importantly, GPX4, which was positively regulated by YAP1 through regulating transcriptional activity, could suppress ferroptosis and induce osteogenic differentiation in OVX-induced mice and osteoblasts. Our findings might provide novel therapeutic therapy targets for delaying OP progression.

## Supplementary Information

Supplementary Material 1.

Supplementary Material 2.

## Data Availability

The data that support the findings of this study are available from the corresponding author upon reasonable request.

## References

[CR1] Ajani JA, et al. YAP1 mediates gastric adenocarcinoma peritoneal metastases that are attenuated by YAP1 inhibition. Gut. 2021;70:55–66. 32345613 10.1136/gutjnl-2019-319748 PMC9832914

[CR2] Compston JE, McClung MR, Leslie WD. Osteoporosis. Lancet. 2019;393:364–76. 30696576 10.1016/S0140-6736(18)32112-3

[CR3] Deng M, et al. METTL14 represses osteoclast formation to ameliorate osteoporosis via enhancing *GPX4* mRNA stability. Environ Toxicol. 2023;38:2057–68. 37195267 10.1002/tox.23829

[CR4] Du YX, Zhao YT, Sun YX, Xu AH. Acid sphingomyelinase mediates ferroptosis induced by high glucose via autophagic degradation of GPX4 in type 2 diabetic osteoporosis. Mol Med. 2023;29:125. 37710183 10.1186/s10020-023-00724-4 PMC10500928

[CR5] Eastell R, et al. Postmenopausal osteoporosis. Nat Rev Dis Primers. 2016;2:16069. 27681935 10.1038/nrdp.2016.69

[CR6] Gao Z, Chen Z, Xiong Z, Liu X. Ferroptosis - a new target of osteoporosis. Exp Gerontol. 2022a;165:111836. 35598699 10.1016/j.exger.2022.111836

[CR7] Gao L, et al. Molecular mechanism of ferroptosis in orthopedic diseases. Cells. 2022b. 10.3390/cells11192979. 36230941 10.3390/cells11192979 PMC9563396

[CR8] Halder G, Johnson RL. Hippo signaling: growth control and beyond. Development. 2011;138:9–22. 21138973 10.1242/dev.045500 PMC2998162

[CR9] Hao J, et al. Qing`e pill inhibits osteoblast ferroptosis via ATM serine/threonine kinase (ATM) and the PI3K/AKT pathway in primary osteoporosis. Front Pharmacol. 2022;13:902102. 35865965 10.3389/fphar.2022.902102 PMC9294279

[CR10] Huang Y, et al. Circular RNA YAP1 attenuates osteoporosis through up-regulation of YAP1 and activation of Wnt/β-catenin pathway. Biomed Pharmacother. 2020;129:110365. 32768931 10.1016/j.biopha.2020.110365

[CR11] Jiang X, Stockwell BR, Conrad M. Ferroptosis: mechanisms, biology and role in disease. Nat Rev Mol Cell Biol. 2021;22:266–82. 33495651 10.1038/s41580-020-00324-8 PMC8142022

[CR12] Jiang Z, et al. Iron overload-induced ferroptosis of osteoblasts inhibits osteogenesis and promotes osteoporosis: an in vitro and in vivo study. IUBMB Life. 2022;74:1052–69. 35638167 10.1002/iub.2656

[CR13] Jing Z, et al. Tobacco toxins induce osteoporosis through ferroptosis. Redox Biol. 2023;67:102922. 37826866 10.1016/j.redox.2023.102922 PMC10571034

[CR14] Ke S, et al. Cytotoxicity analysis of biomass combustion particles in human pulmonary alveolar epithelial cells on an air-liquid interface/dynamic culture platform. Part Fibre Toxicol. 2021;18:31. 34419099 10.1186/s12989-021-00426-x PMC8379799

[CR15] Khosla S, Hofbauer LC. Osteoporosis treatment: recent developments and ongoing challenges. Lancet Diabetes Endocrinol. 2017;5:898–907. 28689769 10.1016/S2213-8587(17)30188-2 PMC5798872

[CR16] Lei SS, et al. Benefits and mechanisms of polysaccharides from Chinese medicinal herbs for anti-osteoporosis therapy: a review. Int J Biol Macromol. 2021;193:1996–2005. 34767882 10.1016/j.ijbiomac.2021.11.030

[CR17] Li H, et al. Mir-484 contributes to diminished ovarian reserve by regulating granulosa cell function via YAP1-mediated mitochondrial function and apoptosis. Int J Biol Sci. 2022;18:1008–21. 35173533 10.7150/ijbs.68028 PMC8771835

[CR18] Li H, et al. 11β-hydroxysteroid dehydrogenase type 1 facilitates osteoporosis by turning on osteoclastogenesis through Hippo signaling. Int J Biol Sci. 2023a;19:3628–39. 37496992 10.7150/ijbs.82933 PMC10367550

[CR19] Li Y, et al. Myoblast-derived exosomal Prrx2 attenuates osteoporosis via transcriptional regulation of lncRNA-MIR22HG to activate Hippo pathway. Mol Med. 2023b;29:54. 37081396 10.1186/s10020-023-00649-y PMC10116833

[CR20] Lin Y, et al. Activation of osteoblast ferroptosis via the METTL3/ASK1-p38 signaling pathway in high glucose and high fat (HGHF)-induced diabetic bone loss. Faseb J. 2022;36:e22147. 35104016 10.1096/fj.202101610R

[CR21] Liu RX, et al. Trim21 depletion alleviates bone loss in osteoporosis via activation of YAP1/β-catenin signaling. Bone Res. 2023;11:56. 37884520 10.1038/s41413-023-00296-3 PMC10603047

[CR22] Lu J, Yang J, Zheng Y, Chen X, Fang S. Extracellular vesicles from endothelial progenitor cells prevent steroid-induced osteoporosis by suppressing the ferroptotic pathway in mouse osteoblasts based on bioinformatics evidence. Sci Rep. 2019;9:16130. 31695092 10.1038/s41598-019-52513-x PMC6834614

[CR23] Ma S, Meng Z, Chen R, Guan KL. The Hippo pathway: biology and pathophysiology. Annu Rev Biochem. 2019;88:577–604. 30566373 10.1146/annurev-biochem-013118-111829

[CR24] Rossini M, Adami G, Adami S, Viapiana O, Gatti D. Safety issues and adverse reactions with osteoporosis management. Expert Opin Drug Saf. 2016;15:321–32. 26699669 10.1517/14740338.2016.1136287

[CR25] Ruan B, et al. DNMT aberration-incurred GPX4 suppression prompts osteoblast ferroptosis and osteoporosis. Bone Res. 2024;12:68. 39617773 10.1038/s41413-024-00365-1 PMC11609303

[CR26] Seibt TM, Proneth B, Conrad M. Role of GPX4 in ferroptosis and its Pharmacological implication. Free Radic Biol Med. 2019;133:144–52. 30219704 10.1016/j.freeradbiomed.2018.09.014

[CR27] Shibata M, et al. Concurrent targeting of potential cancer stem cells regulating pathways sensitizes lung adenocarcinoma to standard chemotherapy. Mol Cancer Ther. 2020;19:2175–85. 32847981 10.1158/1535-7163.MCT-20-0024 PMC7541748

[CR28] Srivastava M, Deal C. Osteoporosis in elderly: prevention and treatment. Clin Geriatr Med. 2002;18:529–55. 12424871 10.1016/s0749-0690(02)00022-8

[CR29] Stockwell BR, Jiang X, Gu W. Emerging mechanisms and disease relevance of ferroptosis. Trends Cell Biol. 2020;30:478–90. 32413317 10.1016/j.tcb.2020.02.009 PMC7230071

[CR30] Wang J, et al. YAP1 protects against septic liver injury via ferroptosis resistance. Cell Biosci. 2022;12:163. 36182901 10.1186/s13578-022-00902-7 PMC9526934

[CR31] Wang Y, et al. Yap1 protects against PM2.5-induced lung toxicity by suppressing pyroptosis and ferroptosis. Ecotoxicol Environ Saf. 2023a;253:114708. 36863160 10.1016/j.ecoenv.2023.114708

[CR32] Wang X, et al. Extracellular vesicles from bone marrow mesenchymal stem cells alleviate osteoporosis in mice through USP7-mediated YAP1 protein stability and the Wnt/β-catenin pathway. Biochem Pharmacol. 2023b;217:115829. 37748664 10.1016/j.bcp.2023.115829

[CR33] Xu P, et al. VDR activation attenuates osteoblastic ferroptosis and senescence by stimulating the Nrf2/GPX4 pathway in age-related osteoporosis. Free Radic Biol Med. 2022;193:720–35. 36402439 10.1016/j.freeradbiomed.2022.11.013

[CR34] Xue Q, et al. Copper-dependent autophagic degradation of GPX4 drives ferroptosis. Autophagy. 2023;19:1982–96. 36622894 10.1080/15548627.2023.2165323 PMC10283421

[CR35] Yan C, et al. Research progress of ferroptosis regulatory network and bone remodeling in osteoporosis. Front Public Health. 2022;10:910675. 35844870 10.3389/fpubh.2022.910675 PMC9280046

[CR36] Yang WS, Stockwell BR. Ferroptosis: death by lipid peroxidation. Trends Cell Biol. 2016;26:165–76. 26653790 10.1016/j.tcb.2015.10.014 PMC4764384

[CR37] Yang WS, et al. Regulation of ferroptotic cancer cell death by GPX4. Cell. 2014;156:317–31. 24439385 10.1016/j.cell.2013.12.010 PMC4076414

[CR38] Yang RZ, et al. Exosomes derived from vascular endothelial cells antagonize glucocorticoid-induced osteoporosis by inhibiting ferritinophagy with resultant limited ferroptosis of osteoblasts. J Cell Physiol. 2021a;236(9):6691–705. 33590921 10.1002/jcp.30331

[CR39] Yuan C, et al. Paeonol protects against acute pancreatitis by inhibiting M1 macrophage polarization via the NLRP3 inflammasomes pathway. Biochem Biophys Res Commun. 2022;600:35–43. 35182973 10.1016/j.bbrc.2022.02.019

[CR40] Yue P, et al. Yap1 modulates cardiomyocyte hypertrophy via impaired mitochondrial biogenesis in response to chronic mechanical stress overload. Theranostics. 2022;12:7009–31. 36276651 10.7150/thno.74563 PMC9576622

[CR41] Zhang J, et al. YAP1 alleviates sepsis-induced acute lung injury via inhibiting ferritinophagy-mediated ferroptosis. Front Immunol. 2022;13:884362. 35979359 10.3389/fimmu.2022.884362 PMC9376389

[CR42] Zhang K, et al. MACF1 overexpression in BMSCs alleviates senile osteoporosis in mice through TCF4/miR-335-5p signaling pathway. J Orthop Transl. 2023;39:177–90. 10.1016/j.jot.2023.02.003 PMC10036500 36969134

[CR43] Zhong D, et al. Circ-itch sponges miR-214 to promote the osteogenic differentiation in osteoporosis via upregulating YAP1. Cell Death Dis. 2021;12:340. 33795657 10.1038/s41419-021-03586-y PMC8016856

