# Supplementary material for: The YAP1/GPX4 axis alleviates osteoporosis by affecting ferroptosis in osteoblasts
Source: Mol Med. 2025 Oct 21;31:315. doi: 10.1186/s10020-025-01374-4 (PMC12539158; doi:10.1186/s10020-025-01374-4)
Supplement: Supplementary file 1 — Supplementary Material 1. [file 10020_2025_1374_MOESM1_ESM.doc]

Supplementary table 1:

| Index | Control （n=28） | OP （n=28） | *p* value |
| --- | --- | --- | --- |
| Age (years) | 53.83±9.44 | 51.94±8.77 | 0.116 |
| BMI (kg/m^2^) | 25.13±2.21 | 24.04±4.56 | 0.260 |
| Smoke (%) | 5(17.86) | 9(32.14) | 0.217 |
| Drink (%) | 8(28.57) | 35 (35.71) | 0.567 |
